# High-Throughput Sequencing-Based Analysis of Rhizosphere and Diazotrophic Bacterial Diversity Among Wild Progenitor and Closely Related Species of Sugarcane (*Saccharum* spp. Inter-Specific Hybrids)

**DOI:** 10.3389/fpls.2022.829337

**Published:** 2022-02-24

**Authors:** Mukesh Kumar Malviya, Chang-Ning Li, Prakash Lakshmanan, Manoj Kumar Solanki, Zhen Wang, Anjali Chandrol Solanki, Qian Nong, Krishan K. Verma, Rajesh Kumar Singh, Pratiksha Singh, Anjney Sharma, Dao-Jun Guo, Eldessoky S. Dessoky, Xiu-Peng Song, Yang-Rui Li

**Affiliations:** ^1^Key Laboratory of Sugarcane Biotechnology and Genetic Improvement (Guangxi), Ministry of Agriculture and Rural Affairs, Sugarcane Research Center, Chinese Academy of Agricultural Sciences, Guangxi Key Laboratory of Sugarcane Genetic Improvement, Sugarcane Research Institute, Guangxi Academy of Agricultural Sciences, Nanning, China; ^2^Interdisciplinary Research Center for Agriculture Green Development in Yangtze River Basin, College of Resources and Environment, Southwest University, Chongqing, China; ^3^Queensland Alliance for Agriculture and Food Innovation, University of Queensland, St. Lucia, QLD, Australia; ^4^Plant Cytogenetics and Molecular Biology Group, Faculty of Natural Sciences, Institute of Biology, Biotechnology and Environmental Protection, University of Silesia in Katowice, Katowice, Poland; ^5^Guangxi Key Laboratory of Agricultural Resources Chemistry and Biotechnology, College of Biology and Pharmacy, Yulin Normal University, Yulin, China; ^6^Department of Agriculture Science, Mansarovar Global University, Bhopal, India; ^7^College of Agriculture, Guangxi University, Nanning, China; ^8^Department of Biology, College of Science, Taif University, Taif, Saudi Arabia

**Keywords:** rhizosphere soil, sugarcane, *nifH*, 16S rRNA, diazotroph diversity

## Abstract

Considering the significant role of genetic background in plant-microbe interactions and that most crop rhizospheric microbial research was focused on cultivars, understanding the diversity of root-associated microbiomes in wild progenitors and closely related crossable species may help to breed better cultivars. This study is aimed to fill a critical knowledge gap on rhizosphere and diazotroph bacterial diversity in the wild progenitors of sugarcane, the essential sugar and the second largest bioenergy crop globally. Using a high-throughput sequencing (HTS) platform, we studied the rhizosphere and diazotroph bacterial community of *Saccharum officinarum* L. cv. Badila (BRS), *Saccharum barberi* (*S*. *barberi*) Jesw. cv Pansahi (PRS), *Saccharum robustum* [*S. robustum;* (RRS), *Saccharum spontaneum* (*S. spontaneum*); SRS], and *Saccharum sinense* (*S. sinense*) Roxb. cv Uba (URS) by sequencing their 16S rRNA and *nifH* genes. HTS results revealed that a total of 6,202 bacteria-specific operational taxonomic units (OTUs) were identified, that were distributed as 107 bacterial groups. Out of that, 31 rhizobacterial families are commonly spread in all five species. With respect to *nifH* gene, *S*. *barberi* and *S. spontaneum* recorded the highest and lowest number of OTUs, respectively. These results were validated by quantitative PCR analysis of both genes. A total of 1,099 OTUs were identified for diazotrophs with a core microbiome of 9 families distributed among all the sugarcane species. The core microbiomes were spread across 20 genera. The increased microbial diversity in the rhizosphere was mainly due to soil physiochemical properties. Most of the genera of rhizobacteria and diazotrophs showed a positive correlation, and few genera negatively correlated with the soil properties. The results showed that sizeable rhizospheric diversity exists across progenitors and close relatives. Still, incidentally, the rhizosphere microbial abundance of progenitors of modern sugarcane was at the lower end of the spectrum, indicating the prospect of *Saccharum* species introgression breeding may further improve nutrient use and disease and stress tolerance of commercial sugarcane. The considerable variation for rhizosphere microbiome seen in *Saccharum* species also provides a knowledge base and an experimental system for studying the evolution of rhizobacteria-host plant association during crop domestication.

## Introduction

Sugarcane is an important agricultural crop grown in nearly 110 countries worldwide. China is the third-largest producer of sugarcane (a collective term for *Saccharum* species, but more commonly applied to cultivated *Saccharum officinarum* (*S. officinarum*) L. and *Saccharum* spp. inter-specific hybrids). It is a major crop in southern China, accounting for ≈90% of Chinese sugar production (Li and Yang, 2015). Over the years, sugarcane has been developed as a multi-purpose agro-industrial crop as it provides the raw material for different industries, such as food, thermal, energy/fuel, and paper (Goldemberg et al., 2008; Fischer et al., 2012). Sugarcane is mainly grown as a monoculture for extended periods resulting in yield decline, which is attributed to degraded soil, imbalanced soil biology, and build-up of pests and diseases (Shoko et al., 2007). Restoration of soil biology and soil fertility is now emerging as a priority for improving soil health, reducing the yield gap, and sustaining profitable green agriculture (Brackin et al., 2013; Schultz et al., 2017).

The rhizosphere is rich in microbial diversity and abundance. These functionally diverse microbial communities include saprophytes, epiphytes, pathogens, and also plant growth-promoting microorganisms (Avis et al., 2008). Bacteria are the most abundant rhizospheric microbiota, covering up to 15% of the total root surface (van Loon, 2007). About 2–5% of rhizobacteria are known to promote plant growth (Antoun and Prévost, 2005). Many plant growth-promoting rhizobacteria (PGPRs) are capable of nitrogen fixation, solubilization of inorganic molecules, such as phosphate, production of plant growth regulators/hormones, siderophores, and compounds that control phytopathogens (Cawoy et al., 2011; Jangu and Sindhu, 2011; Velineni and Brahmaprakash, 2011). Thus, PGPRs and other rhizosphere bacteria are now well-recognized as an essential component of sustainable agriculture systems. The most commonly found rhizospheric bacterial genera are *Bacillus, Pseudomonas, Rhizobia, Arthrobacter, Agrobacterium, Micrococcus, Cellulomonas, Azotobacter, Alcaligenes, Mycobacterium*, and *Flavbacter* (Teixeira et al., 2010; Prashar et al., 2014). There are several PGPRs and diazotrophs genera, such as *Bacillus, Paenibacillus, Pseudomonas, Enterobacter, Arthrobacter, Azotobacter, Burkholderia*, and *Azospirillum*, that are associated with sugarcane rhizosphere (Ahmad et al., 2016; Lamizadeh et al., 2016; Li et al., 2017; Malviya et al., 2019; Pereira et al., 2020; Rosa et al., 2020). The majority of PGPRs are not culturable. Thus studying those using traditional laboratory methods is challenging and time-consuming (Prashar et al., 2014; Wei et al., 2018). Rapid progress in molecular biology, especially the advent of cost-effective, high-throughput DNA sequencing technologies and the associated data analytics, has improved the understanding of rhizosphere microflora by culture-independent studies (Reuter et al., 2015; Wei et al., 2018). The next-generation sequencing approaches provide an efficient and comprehensive system to identify microbial species in the rhizosphere irrespective of microbial abundance (Uroz et al., 2013). As a result, through the sequencing of the 16S rRNA gene, remarkable progress in the taxonomic characterization of highly diversified rhizospheric bacteria has been achieved (Dong et al., 2017; Gong et al., 2019). Further, modern molecular techniques permit an in-depth analysis of soil bacterial communities' compositional and functional dynamics in changing soil environmental conditions, a recurring feature of agricultural soil (Dini-Andreote et al., 2010; Wei et al., 2018). Recently rhizosphere microbiomes community structure of three endangered Plants (Xu et al., 2020) and blueberry varieties (Wang et al., 2019) has been studied by high-throughput sequencing (HTS).

Sugarcane rhizosphere microbes need strong attention to understand their diversity and role in crop improvement. Several novel PGPRs from the sugarcane microbiome have been identified and improved crop production (Pisa et al., 2011; Schultz et al., 2017; Armanhi et al., 2018). Most of the studies on rhizosphere microbes and endophytes are limited to modern commercial sugarcane cultivars, and little is known about their occurrence and abundance in the wild sugarcane progenitor species. They are primarily involved in nitrogen fixation and plant hormone production, thus positively affecting sugarcane growth (Goldemberg et al., 2008; Mehnaz, 2011). The present study focuses on wild sugarcane progenitor species because these species are regarded as high fibrous plants with significant geographic distributions and can survive a broad range of abiotic stresses, such as droughts, saline, floods, and freezing conditions (Santchurn et al., 2019). Rhizobacteria play a significant role in nitrogen fixation in sugarcane crops (Li et al., 2017; Malviya et al., 2019). However, much remains to be learned about these diazotrophic rhizobacteria, a key driver of soil health and fertility. There are several reports of identification and characterization of PGPRs from sugarcane (Inui-Kishi et al., 2012; Lamizadeh et al., 2016) and other crops (Kumar et al., 2014; Gaikwad and Sapre, 2015; Tsegaye et al., 2019) and some of them are being used for crop productivity improvement (Kashyap et al., 2017; Swarnalakshmi et al., 2020). Not surprisingly, most sugarcane studies are conducted with modern cultivated sugarcane hybrid varieties. There is no previous attempt to understand the diversity of rhizobacteria in their wild progenitors and closely related species, such as *S*. *officinarum, Saccharum spontaneum* (*S. spontaneum*)*, Saccharum robustum* (*S. robustum*), *Saccharum barberi* (*S. barberi*), and *Saccharum sinense* (*S. sinense*). Rhizospheric microorganisms interact with plants for their survival and nutritional requirements (Berendsen et al., 2012; Finkel et al., 2017). Many of them are beneficial to plants for nutrient uptake and to cope with pathogens and abiotic stresses (Pisa et al., 2011; Dagnaw et al., 2015; Pereira et al., 2020).

Thus, the current study is aimed to understand the role of rhizosphere bacterial communities and identify new species of nitrogen-fixing bacteria using high-throughput 16S rRNA and *nifH* gene sequencing using the Illumina platform. This study reports novel and valuable findings on the diversity of bacterial communities in five *Saccharum* progenitor species, namely, *S*. *officinarum* L. cv Badila (BRS), *S*. *barberi* Jesw. cv Pansahi (PRS), *S. robustum* (RRS), *S. spontaneum* (SRS), and *S. sinense* Roxb. cv Uba (URS) and provides a knowledge base to study the influence of sugarcane genotype on rhizosphere bacteria in this necessary sugar and energy crop.

## Materials and Methods

### Soil Sampling

**Figure 1:**
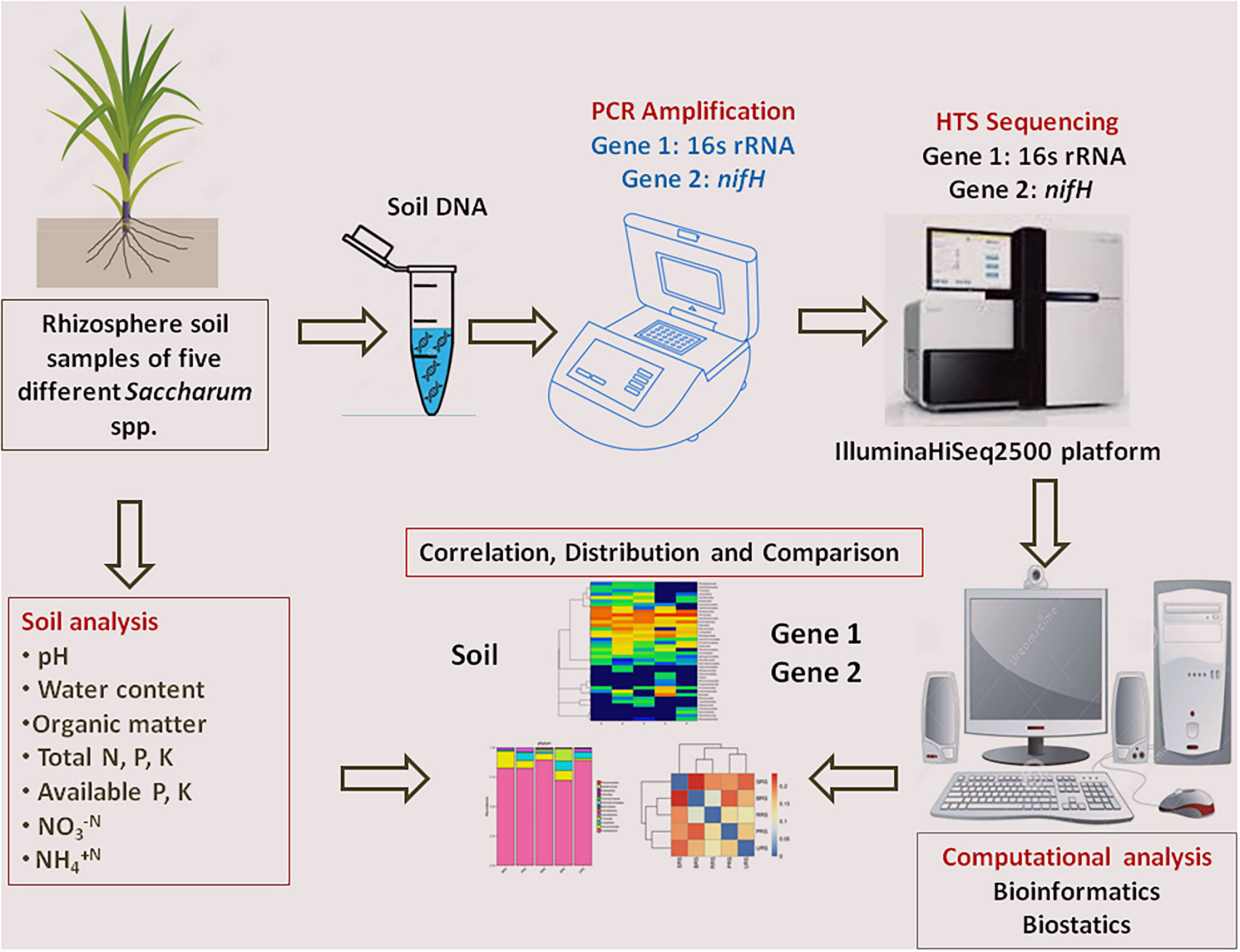
Schematic diagram of experimental design and analysis that used in the current study. HTS, High-throughput sequencing.

Rhizospheric soil of five sugarcane species, BRS, PRS, RRS, SRS, and URS, was maintained in the sugarcane field, germplasm of Sugarcane Research Institute, Guangxi Academy of Agricultural Sciences, Nanning, Guangxi, China (22°49′ N, 108°18′ E, 800–1731 masl). Climate conditions of the site were as follows: natural night (10 h), temperature (minimum 22°C to maximum 35°C), and air humidity was 75–80%. The rhizosphere soil sample of all five sugarcane species was collected at the maturing stage of the first ratoon crop. Five soil samples were separately collected from each species by shaking roots in a sterile bag for 5 min. These soil samples were pooled to prepare a composite soil sample (≈5 g) for each species. Soil DNA from three sub-samples from the composite sample of each species were extracted separately. A schematic diagram represents the overall experimental design (Figure 1).

### Soil Physiochemical Parameters

As described by Solanki et al. (2019), soil chemical analysis activities were carried out. Soil water content was measured by the weighing method. Soil pH was analyzed by a pH meter, and soil organic carbon was measured by the dichromate oxidation method (Walkley and Black, 1934). Total nitrogen (N) was estimated through the semi-micro-Kjeldahl method (Bremmer and Mulvaney, 1982). The FeSO4/Zn reduction method was used for the estimation of nitrate-nitrogen (${\text{NO}}_{3}^{-}$N) and ammonium nitrogen (${\text{NH}}_{4}^{+}$N) (Carter, 1993). Total phosphorus (P) was measured via the sodium carbonate fusion method (Carter, 1993). Available P was estimated by the sample extraction method (Bao, 2002). Total potassium (K) estimation was done by the photometry method (Bao, 2002). The ammonium acetate extraction-flame photometry method was applied to detect the available K in the soil (Bao, 2002). All analysis was performed in three biological replicates.

### High-Throughput Sequencing

A culture-independent method was applied in this study to identify the bacterial composition of test species (Ranjard et al., 2000). Total microbial DNA was extracted using cetyltrimethylammonium bromide/sodium dodecyl sulfate (CTAB/SDS) isolation method with minor modifications (Barbier et al., 2019). The purity of extracted DNA was assayed with a NanoDrop One spectrophotometer (Thermo Fisher Scientific, Wilmington, DE, USA). Three DNA samples were pooled as one and used for DNA sequencing. Bacterial 16S rRNA was amplified with the universal primers 341F (ACTCCTACGGGAGGCAGCAG) and 806R (GGACTACHVGGGTWTCTAAT), which target the V3–V4 regions (Dong et al., 2018). The *nifH* gene was amplified with primers Pol-F (TGCGAYCCSAARGCBGACTC) and Pol-R (ATSGCCATCATYTCRCCGGA), as previously reported (Poly et al., 2001). PCR amplification of identified 16S rRNA and *nifH* genes was performed, and each PCR contains 25 μl reaction that included 12.5 μl ready to use PCR mix (Tiangen Biotech, Beijing, China), 1.0 μl of each primer (10 μM), 2.5 μl of DNA template (10 ng/ml), and 9.0 μl PCR grade water. PCR amplification consisted of a 3 min denaturation at 95°C; 25 cycles of 30 s at 95°C, 30 s at 55°C, and 30 s at 72°C; and 5 min at 72°C for 16S rRNA. For *nifH* gene amplification, thermal cycling consisted of initial denaturation at 98°C for 1 min, followed by 30 cycles of denaturation at 98°C for 10 s, annealing at 50°C for 30 s, and elongation at 72°C for 60 s, finally, 72°C for 5 min, following the protocols reported previously by Zhang et al. (2015). Visualization and quantification of PCR products were conducted by mixing an equal volume of 1 × loading buffer containing SYB green dye with the PCR products and electrophoresed on 2% agarose gel. The 400–450 bp DNA fragments were isolated and used for further experiments. Equimolar amounts of PCR products from all samples were pooled, and the mixture was purified using Qiagen Gel Extraction Kit (Qiagen, Germany). Sequencing libraries of purified amplicons were generated using TruSeq® DNA (Illumina, San Diego, CA, USA). PCR-Free Sample Preparation Kit (Illumina, San Diego, CA, USA) in accordance with the manufacturer's protocol, and index codes were added. The library quality and concentration were assessed on the Qubit@ 2.0 Fluorometer (Thermo Scientific, Waltham, MA, USA) and Agilent Bioanalyzer 2100 system. To perform sequencing, the qualified libraries were fed into the IlluminaHiSeq2500 platform, and 250 bp paired-end reads were generated.

### HTS Data Analysis

Based on their unique barcode, trimming of barcode, and primer sequencing were done. Reads were assembled using FLASH (V.1.2.7) (http://ccb.jhu.edu/software/FLASH/) (Magoè and Salzberg, 2011) to generate raw tags. To obtain high-quality clean tags from raw tags, we performed quality filtration using QIIME (V1.7.0) (http://qiime.org/index.html) (Caporaso et al., 2010). Removal of chimera sequences was done by comparing the tags with the reference database Unite Database (https://unite.ut.ee/) using the UCHIME algorithm (http://www.drive5.com/usearch/manual/uchime_algo.html) (Edgar et al., 2011). The above step is critical to obtain effective tags. Operational taxonomic unit (OTU) identification was done with UPARSE software (v.7.0.1001) (http://drive5.com/uparse/) (Edgar, 2013). Based on ≥95% of sequence similarity, all the effective tags were clustered into OTUs. For each OTU cluster, a representative sequence was screened to perform taxonomic annotation.

Operational Taxonomic Units were taxonomically annotated following a Basic Local Alignment Search Tool (BLAST) analysis against the Unite Database of each identified representative bacterial sequence done in QIIME software. Multiple sequence alignment was conducted with MUSCLE software (V.3.8.3) (http://www.drive5.com/muscle/) (Edgar, 2004), and phylogenetic relationship of different OTUs was established to understand the diversity of microbial species in various samples (groups). Alpha diversity analysis is carried out to find the complexity of species diversity for each sample using 6 indices, which include observed species, Chao1, Shannon, Simpson, abundance-based coverage estimator (ACE), and Good's coverage. Indices calculation for all the samples was done using QIIME and visualized in R software (V. 2.15.3). Community richness was identified with two selected indices using Chao—the Chao1 richness estimator (http://www.mothur.org/wiki/Chao). The Shannon index (http://www.mothur.org/wiki/Shannon) and Simpson index (http://www.mothur.org/wiki/Simpson) indices were used for the identification of community diversity in all the samples. To characterize the sequencing depth and coverage, the Good's coverage (http://www.mothur.org/wiki/Coverage) was used. Beta diversity analysis was performed to evaluate the differences in bacterial species among all the samples. QIIME software was used to calculate beta diversity using weighted and unweighted UniFrac distances. The raw data of 16S rRNA (accession no. PRJNA678588) and *nifH* gene (accession no. PRJNA681283) were submitted to the NCBI Sequence Read Archive.

### Quantitative (q) PCR Analysis

Quantitative PCR was performed to determine the relative abundance of the 16S rRNA and *nifH* gene in each individual rhizospheric soil. The bacterial gene copy numbers were determined with the primers 341F/518R (GC-341 F 5-CCTACGGGAGGCAGCAG-3) and 518 R (5-ATTACCGCGGCTGCTGG-3) (Moore et al., 2011) and the *nifH* gene with the primers *nifH*-F (AAAGGYGGWATCGGYAARTCCACCAC) and *nifH*-R (TTGTTSGCSGCRTACATSGCCAT CAT) (Zhang et al., 2016). Briefly, each 20 μl PCR reaction contained 1.0 μl of DNA template (2.5 ng/μl), 1.0 μl of each primer (10 μmol/l), 7.0 μl of molecular-grade water, and 10 μl iQ™ SYBR Green SuperMix (Bio-Rad Laboratories, Hercules, CA, USA). Conditions of the qPCR for 16S rRNA are as follows: initial denaturation at 95°C for 5 min; 40 cycles of denaturation at 95°C for 15 s, and annealing at 56°C for 30 s. Condition of the qPCR for *nifH* is as follows: 95°C for 30 s followed by 40 cycles of 95°C for 5 s, 60°C for 40 s. The standard curve of DNA and copy number was constructed by the standard formula: y = −3.406 × 37.05 [y: Ct value; × LOG10 (copy number)]. All samples were done in three replicates.

### Statistical Analysis

Using the FactoMineR and ggplot2 packages in R software, results were visualized. Later, Principal Coordinate Analysis (PCoA) was performed to obtain principal coordinates and to visualize them. To compare microbial diversity in different samples, the UniFrac method was used to generate weighted or unweighted UniFrac values among samples that were transformed to give uncorrelated/orthogonal axes. Visualization of PCoA results was done using weighted gene co-expression network analysis (WGCNA) package, stat, and ggplot2 packages in R software. QIIME software was used for hierarchical clustering by unweighted pair-group method with arithmetic mean method (UPGMA) (Sokal, 1958) to infer the distance matrix using average linkage. Heatmaps and Venn plots were generated using the package “ggplots” of software R (v3.0.3). The experiments were conducted in replicates, and data were analyzed using standard ANOVA followed by the Duncan's Multiple Range Test (DMRT) through Origin 2017SR2 software (Northampton, MA, USA). Circos plots were drawn by Circos Table Viewer v0.63-9 software (Krzywinski et al., 2009) to calculate Spearman's rank correlation coefficient between soil variables and bacterial taxa by using PAST3 software (Hammer et al., 2001), and heatmap is generated by using ClustVis online tool (Metsalu and Vilo, 2015) and Morpheus (https://software.broadinstitute.org/morpheus/).

## Results

This study was aimed to understand the rhizosphere soil microbiota of five different critical ancestral sugarcane species using 16S rRNA and *nifH* gene sequencing to understand their bacterial community diversity, especially that of diazotrophs. This is a major knowledge gap as the significance of rhizosphere biology has a strong genetic basis. It is now increasingly recognized as a target for crop productivity improvement in all major crops, such as sugarcane.

### Soil Physiochemical Parameters

**Figure 2:**
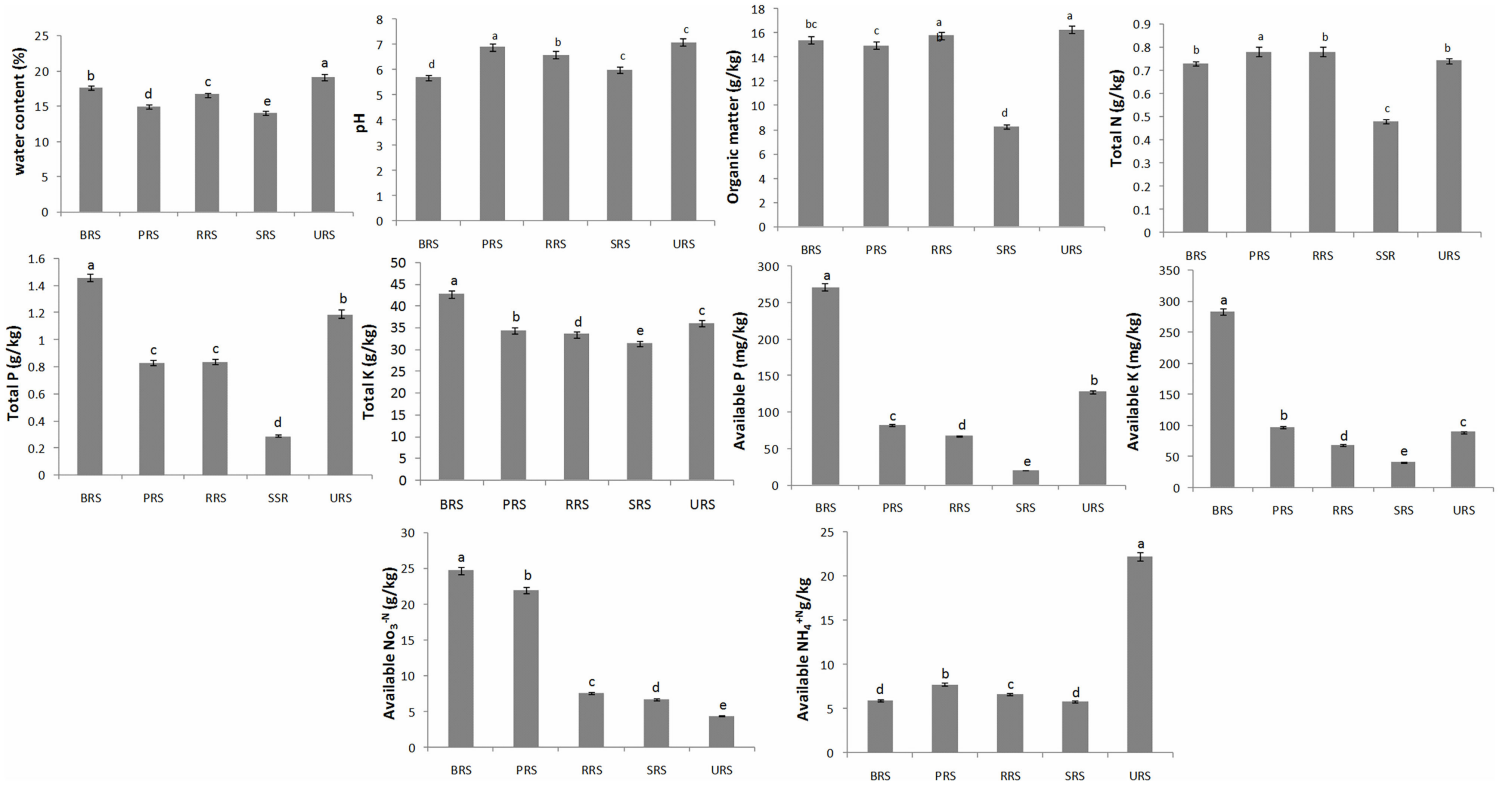
Soil physiochemical properties of rhizospheric soil samples of sugarcane species. Values followed by the same lowercase letters in the same bar are not significantly different according to DMRT 5%. *S*. *officinarum* L. cv Badila (BRS), *S*. *barberi* Jesw. cv Pansahi (PRS), *S. robustum* (RRS), *S. spontaneum* (SRS), and *S. sinense* Roxb. cv Uba (URS). DMRT, Duncan's Multiple Range test.

The physiochemical properties of all the rhizosphere soil samples are presented in Figure 2. Higher water content values were found in *S. sinense* (19.1 ± 0.38%), and the low values were found in *S. spontaneum* (14% ± 0.33). The pH range was recorded (*S. sinense*) 7.1 ± 0.14 to 5.7 ± 0.11 (*S*. *officinarum*). Organic matter (OM) was observed higher in *S. sinense* (16.30 ± 0.33) and lowest in *S. spontaneum* (8.29 ± 0.17). The higher amounts of total N were recorded in *S*. *barberi* (0.78 ± 0.02), and low quantity resulted in *S. spontaneum* (0.48 ± 0.11). Total K, available P, and available K were recorded higher in *S*. *officinarum* and low in *S. spontaneum*. The higher value of available ${\text{NO}}_{3}^{-}$N was observed in *S*. *officinarum* (24.70 ± 0.49) and the lowest value in *S. sinense* (4.43 ± 0.09). A higher concentration of available ${\text{NH}}_{4}^{+}$N was recorded in *S. sinense* (22.20 ± 0.44) and low in *S. spontaneum* (5.79 ± 0.12).

### Sequencing Data

A total of 233,455 effective sequences with an average length of 413 bp were obtained for 16S rRNA genes from different sugarcane species samples. Sequencing of *nifH* genes from five different species samples produced a total of 182,185 sequences with 357 average bp length. These raw reads of 16S rRNA and nifH genes were filtered using QIIME quality filters, followed by OTU identification, clustering, and analysis, respectively (Supplementary Tables S1, S2).

### Operational Taxonomic Units

**Figure 3:**
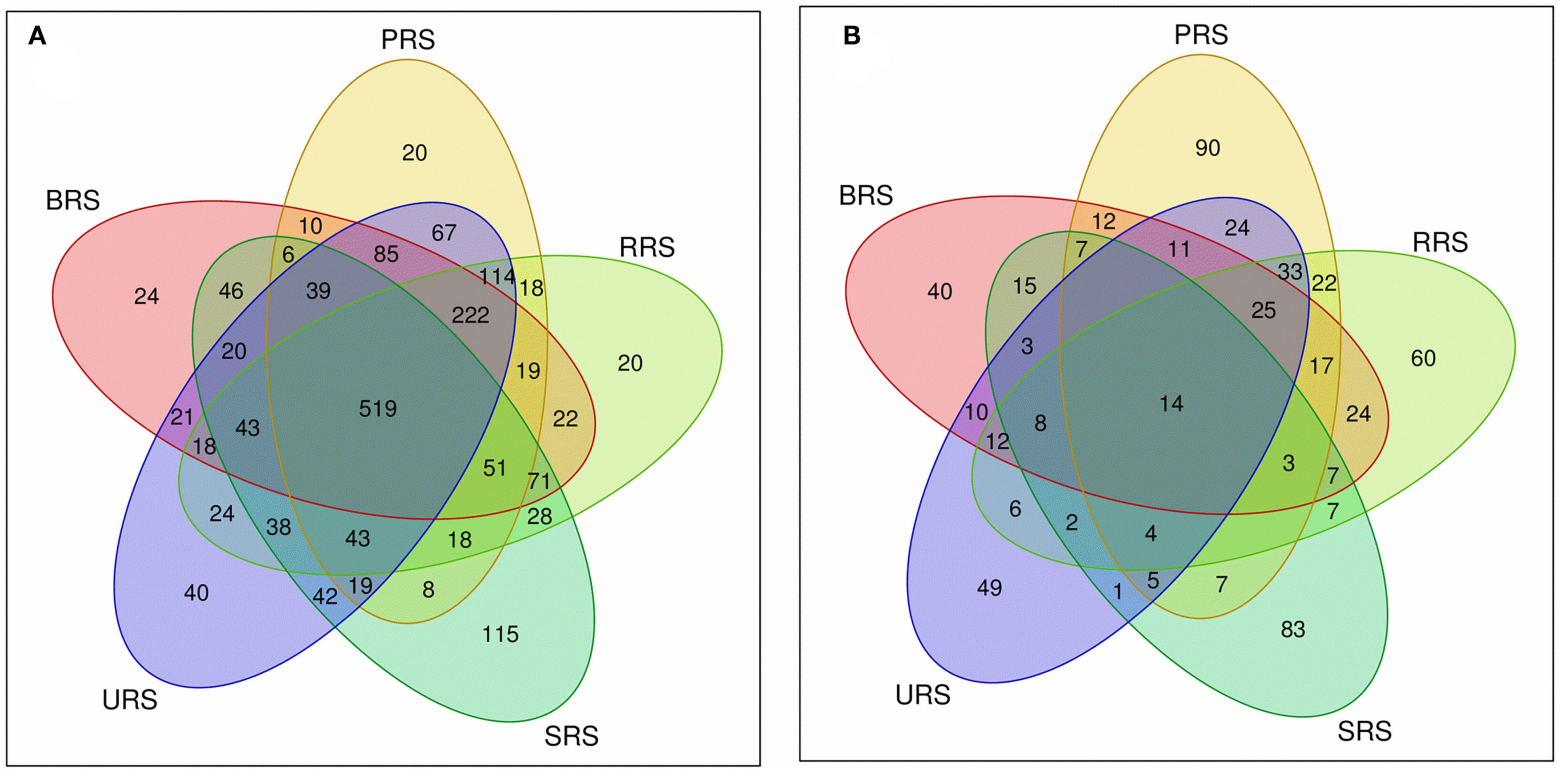
Venn diagram showing the OTUs distribution between the sugarcane species soil samples **(A)** 16S rRNA and **(B)** *nifH* gene. *S*. *officinarum* L. cv Badila (BRS), *S*. *barberi* Jesw. cv Pansahi (PRS), *S. robustum* (RRS), *S. spontaneum* (SRS), and *S. sinense* Roxb. cv Uba (URS). OTUs, operational taxonomic units.

The *S. spontaneum* sample, compared to those from all the other species, showed less species evenness at a low OTU rank. We analyzed common and unique OTUs based on 16S rRNA and *nifH* gene sequences for each sample (Supplementary Figures S1A,B). In the 16S rRNA sequence data, 6202 OTUs were identified from all samples collectively, of which 519 OTUs were common across all species. The relative frequency of OTUs in the studied species was as follows: *S. sinense* > *S. robustum* > *S*. *barberi* > *S*. *officinarum* > *S. spontaneum* (Figure 3A). A total of 1,099 OTUs were identified for *nifH* gene from the sequence data of species combined. Among them, 14 were common OTUs found across all the samples. The occurrence of OTUs in the plant samples was as follows: *S*. *barberi* > *S. robustum* > *S*. *officinarum* > *S. sinense* > *S. spontaneum*. Thus, *S*. *barberi* and *S. spontaneum* recorded the highest and lowest number of OTUs (Figure 3B).

### Principal Component Analysis (PCA)

To understand the rhizobacterial community composition, PCA plots were generated based on 16S rRNA and *nifH* gene OTUs data (Supplementary Figures S2A,B). The OTUs of 16S rRNA samples show that *S*. *officinarum* and *S. robustum* are not identical, *S. sinense* and *S. robustum* have more similarity, and *S. spontaneum* is distinct from all the other species studied. PCA of *nifH* gene samples showed a close identity between *S*. *barberi* and *S. spontaneum*, whereas *S. sinense, S*. *officinarum*, and *S. robustum* remained distinct.

### Diversity Index and Microbial Composition

Alpha diversity refers to the diversity within a particular sample individually, and it is usually represented by the microbial species (i.e., species richness) enumerated in the test samples. Alpha diversity analysis was done using Shannon, Simpson, and Chao indices Rarefaction curves for both 16S rRNA and *nifH* sequence data. Supplementary Figure S3A consists of plots displaying Shannon, Simpson, and Chao indices, built using 16S rRNA samples. The Shannon index was increased as both the species richness and the evenness in the community was increased. Among the 16S rRNA data of all the samples analyzed, the Shannon and Chao indices of *S. sinense* sample were higher than the other four samples, with a slight variation found among them. Whereas, Simpson indices was increased as the diversity was increased. In the Simpson index plot, *S*. *officinarum* samples showed the highest value, implying more species diversity than the other four samples. Shannon and Chao indices of *nifH* gene data of all the samples showed more species diversity in *S*. *barberi* sample than others. Like 16S rRNA data, *S*. *officinarum* sample showed the highest value in Simpson index based on *nifH* gene data (Supplementary Figure S3B).

**Figure 4:**
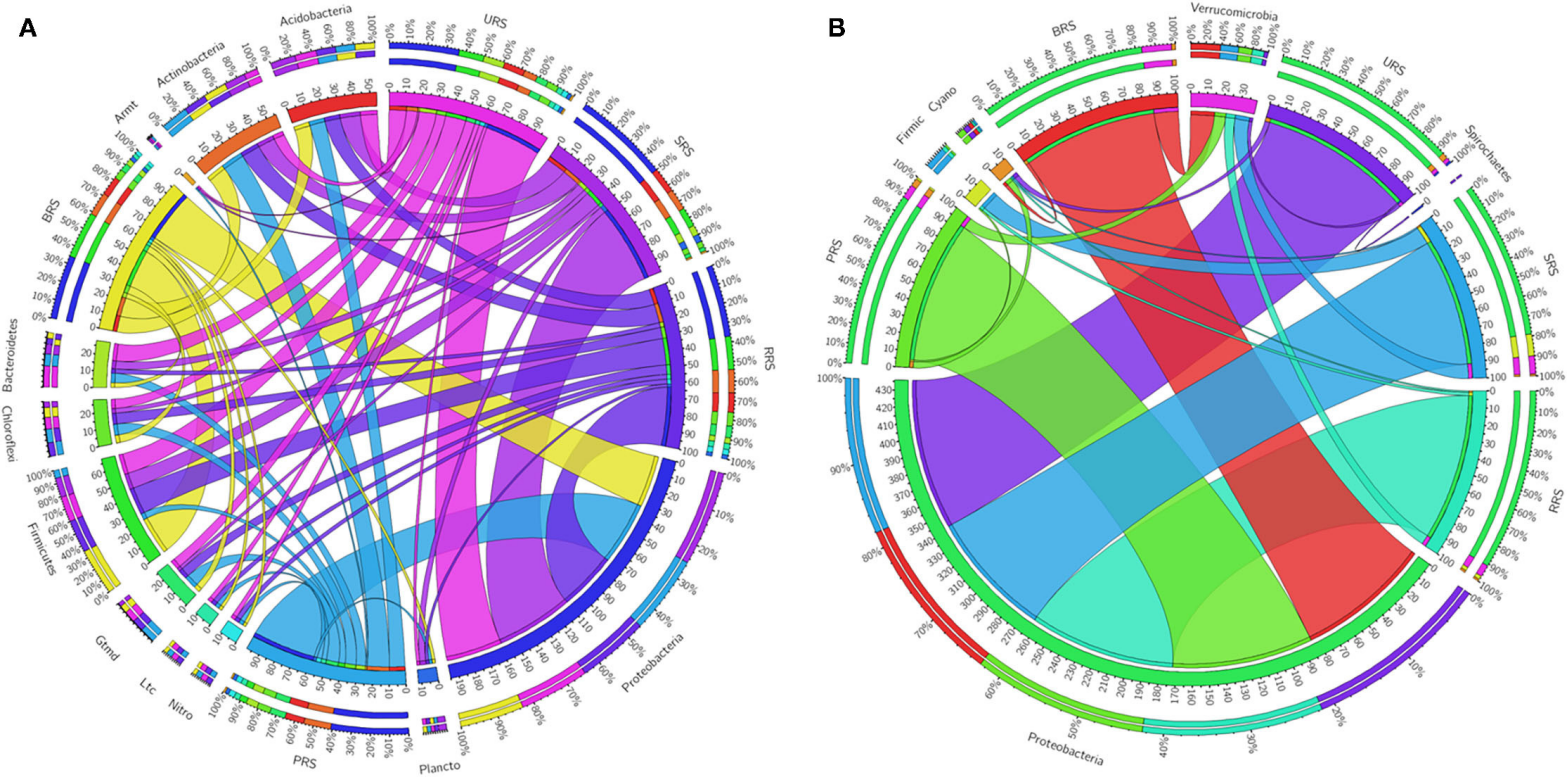
Circular representation of the proportional structure of bacterial communities at the phylum level based on 16s rRNA **(A)** and *nifH* gene **(B)** associated with the sugarcane rhizosphere of different species. Values within the inner circle indicate the number of reads of a phylum within the normalized dataset. *S*. *officinarum* L. cv Badila (BRS), *S*. *barberi* Jesw. cv Pansahi (PRS), *S. robustum* (RRS), *S. spontaneum* (SRS), and *S. sinense* Roxb. cv Uba (URS).

The relative abundance of the microbial communalities was differed among the five sugarcane species analyzed. The abundant phyla identified in all sugarcane species using 16S rRNA data were Proteobacteria, Firmicutes, Actinobacteria, Acidobacter, Bacteroidetes, Chloroflexi, Gemmatimonadetes, Planctomycetes, and Nitrospirae (Figure 4A). Firmicutes were the highest phyla present in *S*. *officinarum* compared to other samples. Gemmatimonadetes were the highest in *S*. *barberi*, Acidobacter was more elevated in *S. spontaneum*, and Bacteroidetes was most increased in *S. sinense*. Based on *nifH* gene data, we found Proteobacteria and Verrucomicrobia (Figure 4B). Firmicutes were abundant in *S. spontaneum* samples compared to others. Many unclassified phyla were also represented abundantly in *S. spontaneum* followed by *S. sinense* and *S*. *barberi* samples.

**Figure 5:**
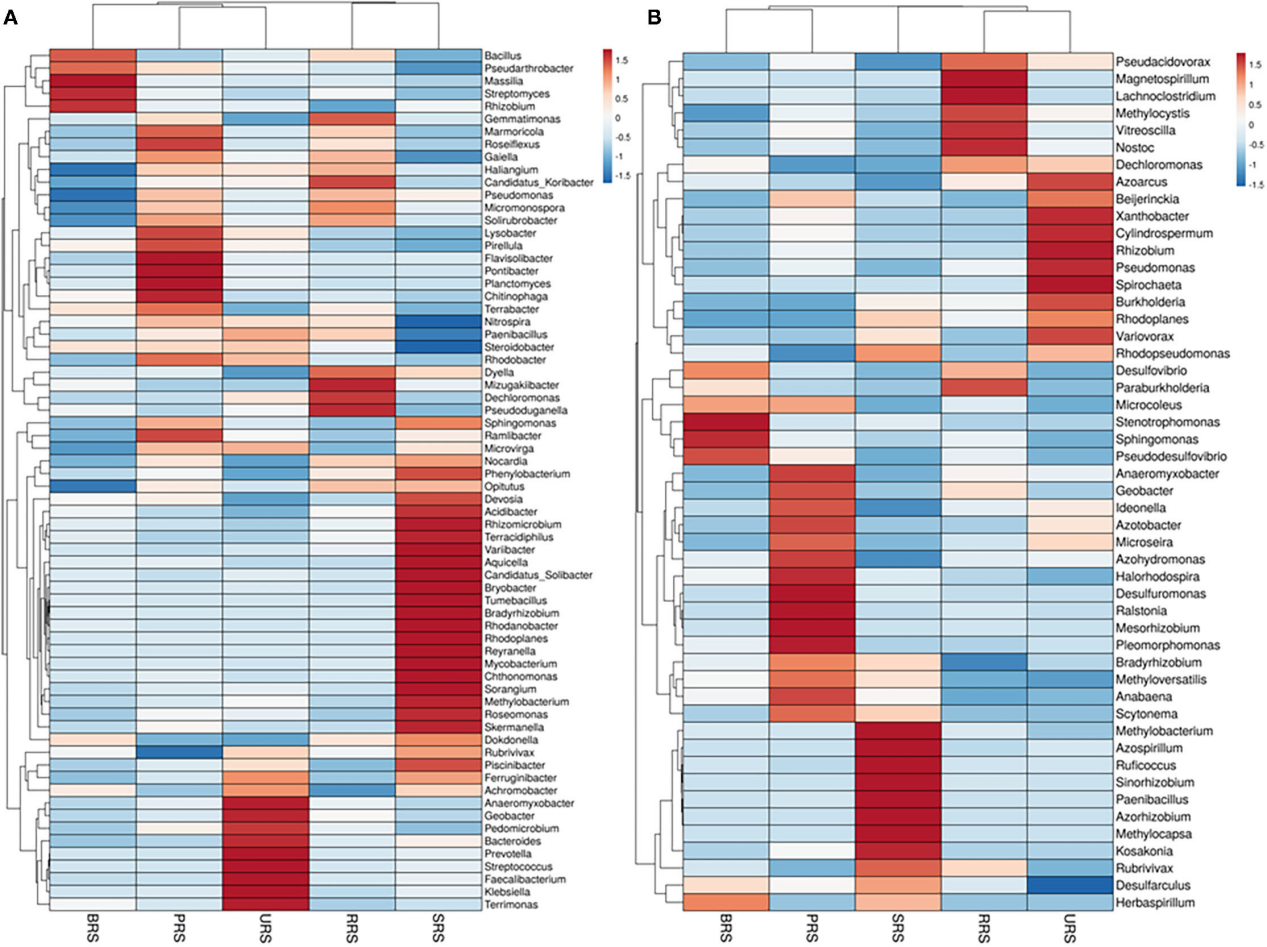
Heat map showing the abundances of bacterial communities at genus level in the soil samples of sugarcane species **(A)** 16S rRNA and **(B)** *nifH* gene. *S*. *officinarum* L. cv Badila (BRS), *S*. *barberi* Jesw. cv Pansahi (PRS), *S. robustum* (RRS), *S. spontaneum* (SRS), and *S. sinense* Roxb. cv Uba (URS).

Genus distribution using 16S rRNA sequence data is given in Figure 5A. *Bacillus* was the most abundant genus in *S*. *officinarum, S. robustum*, and *S. sinense*, while *Pseudomonas* became the number one genus in *S*. *barberi* and *S. spontaneum*. *Bacillus, Pseudomonas, Pseudarthobacter, Massilia, Lysobacter, Nitrospira, Gemmatimonas*, and *Streptomyces*, and *Rhizobium* were the most abundant in *S*. *officinarum*. However, *S*. *barberi* soil sample was dominated by *Bacillus, Pseudomonas, Pseudarthobacter, Lysobacter, Gemmatimonas*, and *Sphingomonas*. *S. robustum* contained *Bacillus, Pseudomonas, Pseudarthobacter, Massilia, Nitrospira, Gemmatimonas, Streptomyces, Paenibacillus*, and *Dechloromonas*. *S. spontaneum* sample contained *Pseudomonas, Pseudarthobacter, Massilia, Tumebacillus, Remibacter, Sphingomonas*, and *Skermanelia. S. sinense* sample was dominated by *Bacillus, Pseudomonas, Pseudarthobacter, Massilia, Lysobacter, Nitrospira, Faecalibacterium*, and *Streptococus*.

Genus distribution using *nifH* gene data is presented in Figure 5B. *Bradyrhizobium, Dechloromonas, Desulfovibrio*, and *Stenotrophomonas* were abundant in *S*. *officinarum*, while *Bradyrhizobium, Desulfovibrio, Xanthobacter*, and *Anaeromyxobacter* were the leading genera in *S*. *barberi*. *Bradyrhizobium, Dechloromonas, Desulfovibrio*, and *Anaeromyxobacter* were the dominant groups in *S. robustum. S. spontaneum* contained *Bradyrhizobium, Azospirillum, Methanobacterium*, and *Paenibacilllus* species. *Bradyrhizobium, Dechloromonas, Xanthobacter* and *Anaeromyxobacter* were present in *S. sinense*. Genus *Burkolderia* was found in *S*. *barberi, S. spontaneum*, and *S. sinense* while *Beijerinckia* was recorded in *S*. *barberi, S. spontaneum*, and *S. sinense*. Genus *Idenella* was commonly present in all the samples except *S. spontaneum*, and *Kosakonia* was normally present in *S*. *barberi and S. spontaneum*.

**Figure 6:**
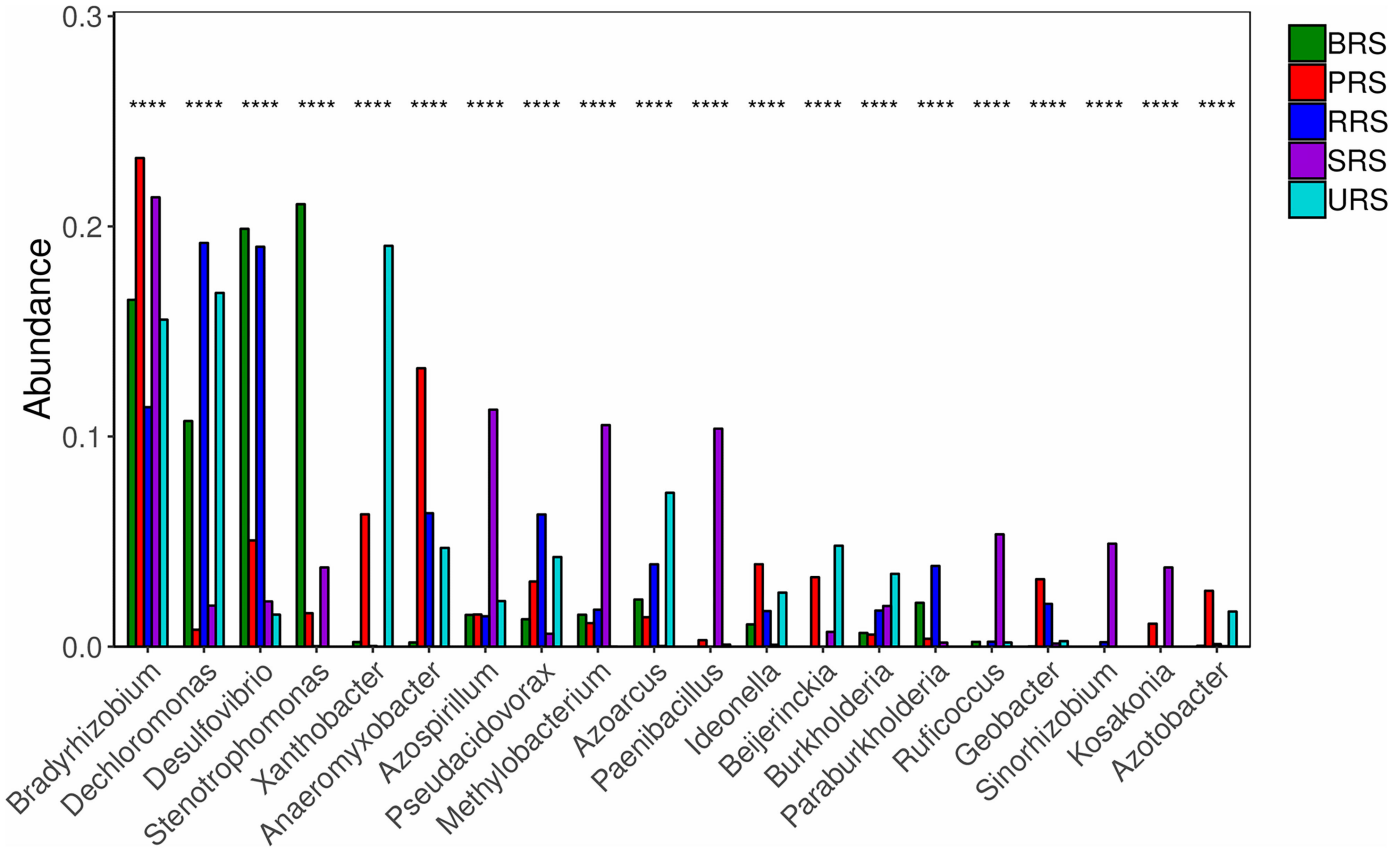
The relative abundances of the top 20 diazotrophs at the genus level in the soil of sugarcane species. *S*. *officinarum* L. cv Badila (BRS), *S*. *barberi* Jesw. cv Pansahi (PRS), *S. robustum* (RRS), *S. spontaneum* (SRS), and *S. sinense* Roxb. cv Uba (URS). Note: Select species with *p* < 0.05 to draw a column chart, each column is the relative abundance of different species, where **** means *p* < 0.0001.

Star analysis was conducted using the top 10 genera of each sample. The top 10 bacterial genera of 16S rRNA data-based analysis were *Pseudarthobacter, Pseudomonas, Bacillus, Massilia, Gemmatimonas, Nitrospora, Haliangium, Ramibacter, Tumebacillus*, and *Lysobacter*. From this analysis, the *Bacillus* genus was the leading one in *S*. *officinarum* and *S. robustum* samples, while *Pseudomonas* was the dominant one in *S*. *barberi, S. robustum, S. spontaneum*, and *S. sinense. Tumebacillus* genus was found only in *S. spontaneum* (Supplementary Figure S4A). Similarly, star analysis for *nifH* gene samples identified the presence of *Bacillus* in all the samples. *Desulfovibrio* was found in significant numbers *in S. officinarum, S. robustum*, and *S. barberi*. *Xanthobacter* was abundant in *S. sinense. Anaeromyxobacter* was found in all the samples except that of *S. spontaneum. Pseudoacidovorax* genus was found only in *S. robustum, S*. *barberi*, and *S. sinense* samples. *Azospirillum* and *Methylobacterium* were unique to *S. spontaneum* (Supplementary Figure S4B). The relative abundance of the top 20 diazotrophs at the genus level present in all sugarcane species is shown in Figure 6. Diazotrophs belonging to the genus *Bradyrhizobium* were present in all the samples tested.

### Beta Diversity

Beta diversity measures species diversity among different samples collected from similar or different environments. We performed the PCoA and UniFrac-based cluster analysis to understand the beta diversity of 16S rRNA and *nifH* gene in the sugarcane species studied (Supplementary Figures S5, S6). Based on 16S rRNA data, *S. sinense*, along with *S*. *barberi*, and *S*. *officinarum*, along with *S. robustum*, formed independent clusters. Moreover, *S. spontaneum* was segregated away from all others, displaying higher beta diversity (Supplementary Figure S5A). Similarly based on *nifH* data, *S. spontaneum* was segregated away from all others samples (Supplementary Figure S6A). Hierarchical clustering based on the UniFrac cluster analysis showed similar results for both 16S rRNA and *nifH* gene data, containing identical sequences showing 0 (blue color) distances. *S. sinense* to *S. spontaneum* showed more distance (red color = 0.3), indicating dissimilarity in sequences of *S. sinense* to *S. spontaneum* samples (Supplementary Figures S5b, S6b).

**Figure 7:**
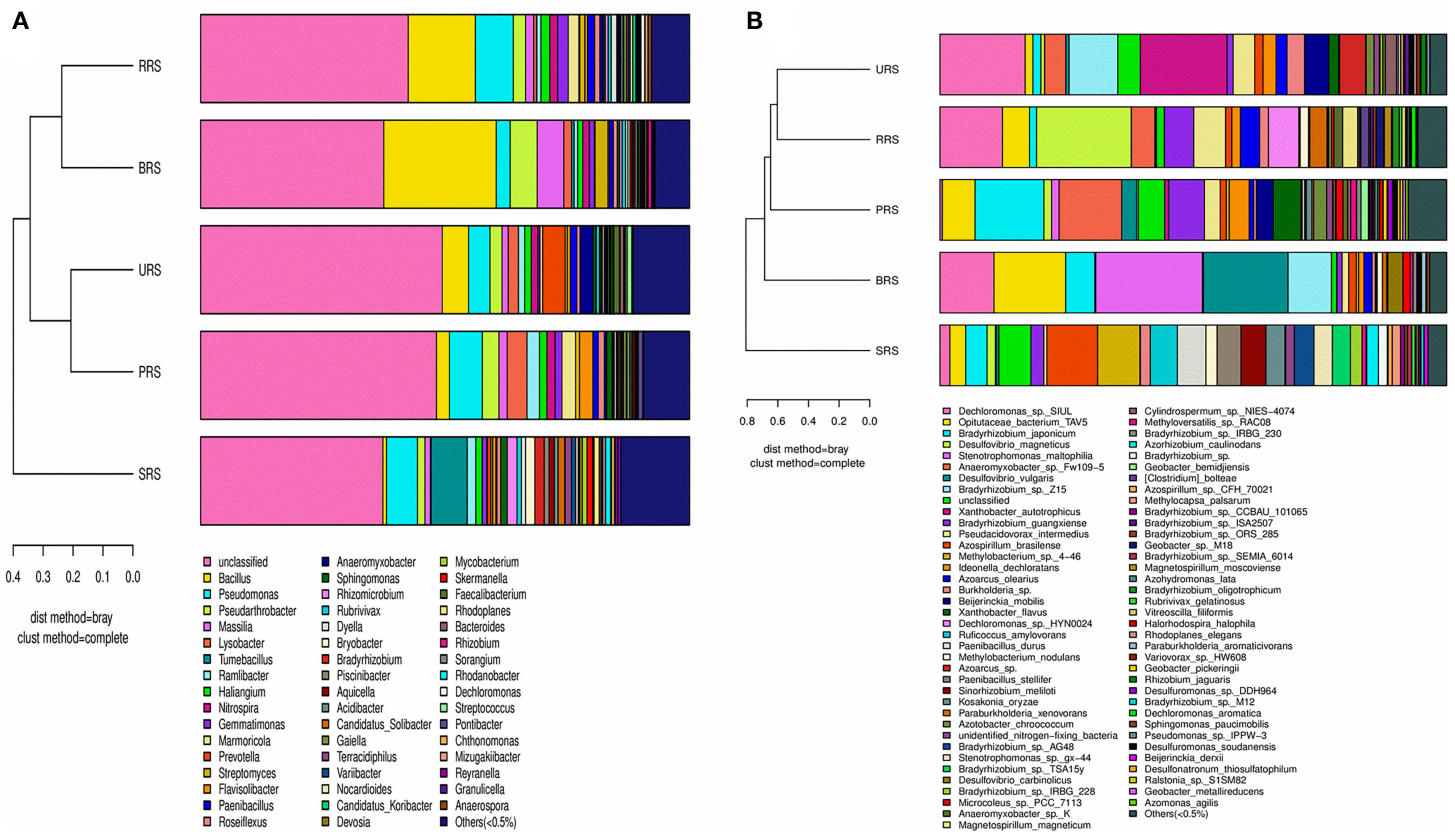
A combination of horizontal multi-sample similarity trees and histograms 16S rRNA **(A)** and *nifH* genes **(B)**. On the left is hierarchical clustering between samples based on community composition (Bray-Curtis algorithm), and on the right is a column chart of the sample's community structure. *S*. *officinarum* L. cv Badila (BRS), *S*. *barberi* Jesw. cv Pansahi (PRS), *S. robustum* (RRS), *S. spontaneum* (SRS), and *S. sinense* Roxb. cv Uba (URS).

The distribution of dominant genera based on their relative abundance performed with the Bray-Curtis algorithm showed *Bacillus* being the dominant genus in all the samples except those from *S. spontaneum* (Figure 7A). *Pseudomonas* was the second dominant genus, while *Pseudarthobacter* became the third leading one. *S. spontaneum* had most of the unidentified genera in our analysis. Similarly, the distribution of dominant genera in *nifH* samples showed the presence of *Dechloromonas* spp. in all samples except those from *S*. *barberi*. *Xanthobacter* and *Bradyrhizobium* were found to be prevalent in *S*. *officinarum* and *S. sinense*, respectively (Figure 7B).

### qPCR

**Figure 8:**
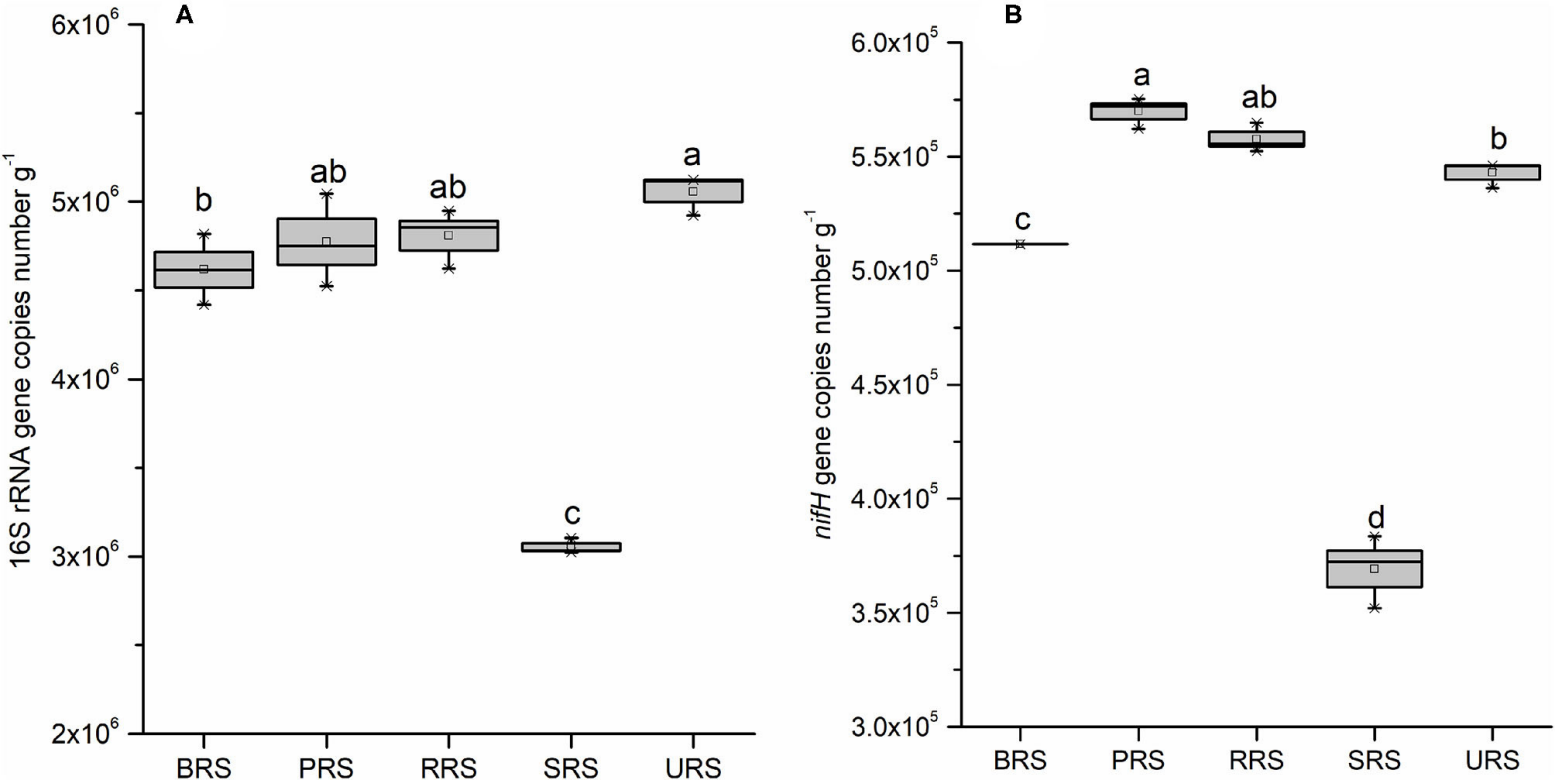
Box plot estimated values of gene copies of 16S rRNA **(A)**, and *nifH* **(B)** in the soil samples of sugarcane species. Within each box, Small squares denote mean values; boxes extend from the 10th to the 90th percentile of each group's distribution of values. Values followed by the same lowercase letters in the same column are not significantly different according to DMRT 5%. DMRT, Duncan's Multiple Range Test.

To quantify bacterial abundance in all five species, we performed qPCR analysis using the 16S rRNA and *nifH* gene-specific primer (Figure 8). Occurrences of gene copy number, indicating bacterial abundance, in all species were as follows: *S. sinense* > *S. robustum* > *S*. *barberi* > *S*. *officinarum* > *S. spontaneum* (Figure 8A). The *nifH* gene copy number results showed that N-fixing bacterial population was highest in *S*. *barberi* followed by *S. robustum* > *S*. *officinarum* > *S. sinense* with the least in *S. spontaneum* (Figure 8B). Thus, *S*. *sinense* had the most considerable rhizospheric bacterial abundance, while N-fixing bacteria were most prolific in the rhizosphere of *S. barberi*.

### Spearman's Rank Correlation

**Figure 9:**
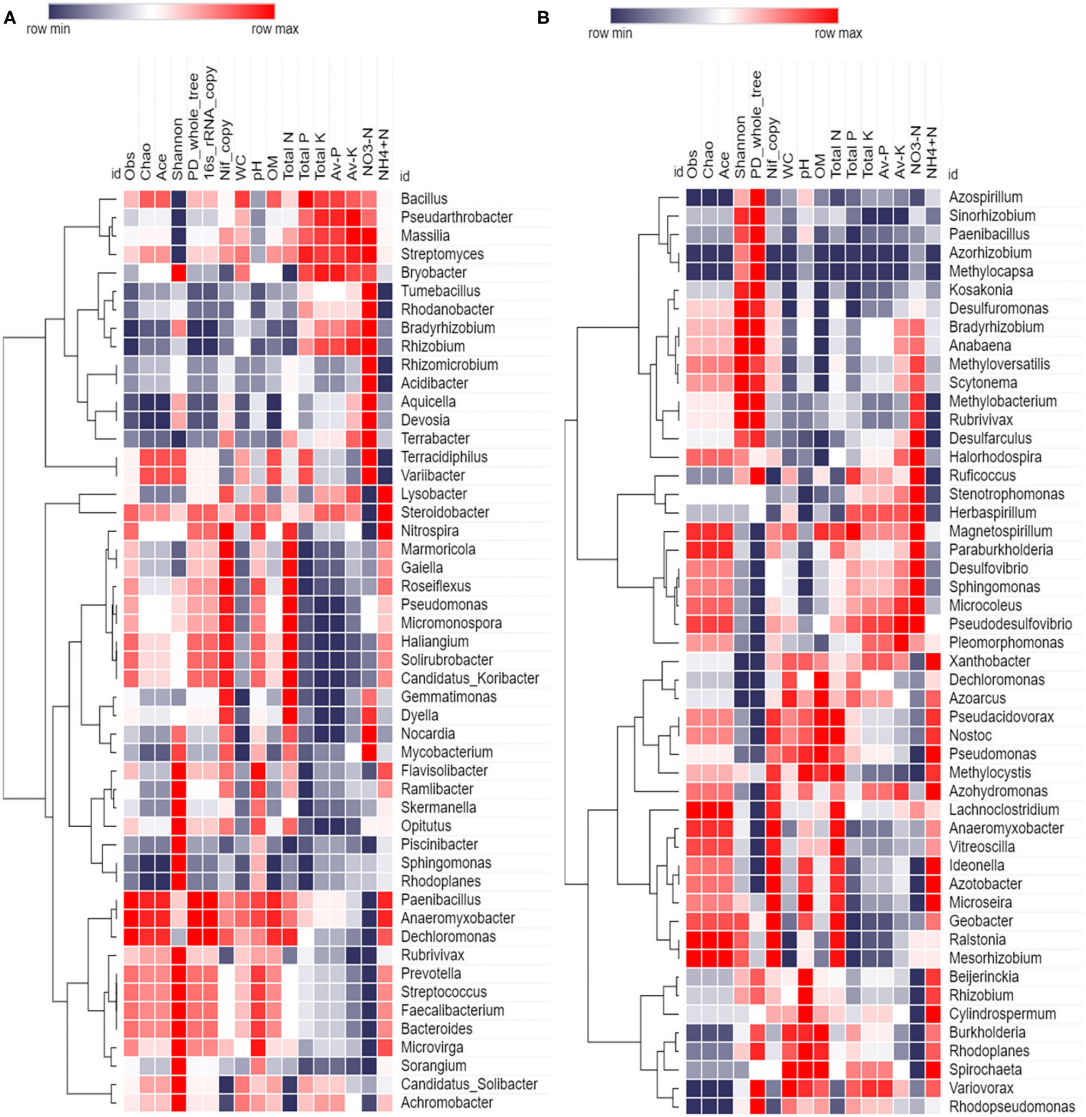
Spearman correlation heatmap based on abundance (top 50 genera), diversity indices, gene copy, and soil variables **(A)** 16S rRNA **(B)** *nifH* gene.

Spearman rank correlation analysis was calculated based on 16S rRNA and *nifH* gene abundance (top 50), diversity indices, gene copy, and soil variables; and values were illuminated in a heat map (Figure 9). Spearman's rank correlation coefficients were calculated to assess the association between soil physiochemical properties and dominant (16S rRNA and *nifH*) bacterial genera. The relationships were estimated between these taxa and physiochemical properties to understand the role of the microbial community shift. Related heat maps show that soil physiochemical factors significantly affected the relative abundance of bacterial taxa (phyla and genera). 16S rRNA bacterial abundance shows a strong positive correlation (*r* = 0.8, *p* < 0.1) between pH and abundance of bacteria, such as *Nitrospira, Flavisolibacter*, and *Paenibacillus*. OM had a positive correlation (*p* < 0.05) with *Paenibacillus, Anaeromyxobactor*, and *Microvirga*. Total N had a positive correlation (*r* = 0.9, *p* < 0.05) with *Marmoricola* and *Gaiella*. Total P had positive correlation (*p* < 0.05) with *Bacillus* (*r* = 0.9) and *Streptomyces* (*r* = 0.6). Total K had positive correlation (*r* = 0.9, *p* < 0.01) with *Pseudarthobacter* and *Streptomyces*. Available P had positive correlation with *Pseudarthobacter* (*r* = 0.9, *p* < 0.05). While, available K had positive correlation with *Pseudarthobacter* (*r* = 1, *p* < 0.05) and *Massilia* (*r* = 0.9, *p* < 0.05). Moreover, available ${\text{NO}}_{3}^{-}$N showed a positive correlation with *Massilia* (*r* = 0.9, *p* < 0.05), *Terrabacter* (*r* = 0.9, *p* < 0.5), and *Streptomyces* (*r* = 0.8, *p* < 0.1). Available ${\text{NH}}_{4}^{+}$N had a positive correlation with *Lysobacter* (*r* = 0.8, *p* < 0.1), *Nitrospira* (*r* = 0.9, *p* < 0.05), *Paenibacillus* (*r* = 0.9, *p* < 0.05), *Anaeromyxobacter* (*r* = 0.9, *p* < 0.05), and *Steroidobacter* (*r* = 0.9, *p* < 0.05). *Paenibacillus* and *Anaeromyxobacter* strongly correlated with obs, Chao, PD_whole tree, and OM. However, *Nocardia* had a significant negative correlation with total K, while *Tumebacillus* showed a negative correlation with pH. In the case of *nifH* gene, diversity indices (observed species, Ace, Chao) had a strong positive correlation with *Anaeromyxobacter* (*r* = 0.9, *p* < 0.05), *Lachnoclostridium* (*r* = 0.9, *p* < 0.05), and *Vitreoscilla* (*r* = 0.8, *p* < 0.1). The qRT-PCR data (*nifH* gene copy number) had a strong positive correlation (*p* < 0.5) with *Anaeromyxobacter, Ideonella, Geobacter, Azotobacter*, and *Vitreoscilla*. WC had positive correlation with *Azoarcus* (*r* = 0.9, *p* < 0.05), and soil pH showed a strong positive correlation with *Ideonella* (*r* = 0.8, *p* < 0.1), *Azotobacter* (*r* = 0.8, *p* < 0.1), *Rhizobium* (*r* = 0.9, *p* < 0.05), and *Pseudomonas* (*r* = 0.8, *p* < 0.1). Similarly, soil OM had a strong positive correlation with *Azoarus* (*r* = 1, *p* < 0.05), *Pseudomonas* (*r* = 0.9, *p* < 0.05), and *Nostoc* (*r* = 0.8, *p* < 0.1). Soil total N revealed a strong positive correlation with *Vitreoscilla* (*r* = 0.9, *p* < 0.05), and available K showed with *Pseudosulfovibrio* (*r* = 0.9, *p* < 0.05). While, soil available ${\text{NO}}_{3}^{-}$N was correlated positively to *Desulfovibrio* (*r* = 0.9, *p* < 0.05), *Microcoleus* (*r* = 0.97, *p* < 0.05), *Halorhodospira* (*r* = 0.8, *p* < 0.1), *Sphingnomonas* (*r* = 0.9, *p* < 0.05), and *Pseudosulfovibrio* (*r* = 0.9, *p* < 0.05). Moreover, soil available ${\text{NH}}_{4}^{+}$N showed strong correlation with *Xanthobacter* (*r* = 0.9, *p* < 0.05), *Ideonella* (*r* = 0.9, *p* < 0.05), *Azotobacter* (*r* = 0.9, *p* < 0.05), *Pseudomonas* (*r* = 0.9, *p* < 0.05), and *Azohydromonas* (*r* = 0.08, *p* < 0.1). However, pH was correlated significantly negative with the *Stenotrophomonas* (*r* = −0.9, *p* < 0.05), and OM was correlated strongly negative with *Desulfarculus* (*r* = −0.9, *p* < 0.05) and *Anabaena* (*r* = −0.9, *p* < 0.1). Similarly, soil available ${\text{NH}}_{4}^{+}$N showed a negative correlation with *Methylobacter* (*r* = −0.9, *p* < 0.05) and *Desulfarculus* (*r* = −0.9, *p* < 0.05).

## Discussion

The present study focuses on wild sugarcane progenitors because these species are distributed in different geographic distributions and service in various biotic and abiotic stresses (Santchurn et al., 2019). To understand the PGPRs and nitrogen-fixing diazotrophs, we need to study the sugarcane rhizosphere microbiome community. In most crops, such as sugarcane, a large proportion of PGPRs are yet to be identified. Bacteria are the most abundant of all the rhizospheric microbiota, and many are known to promote plant growth (Antoun and Prévost, 2005; van Loon, 2007; Dagnaw et al., 2015). The present study made an effort to understand the diverse and complex bacterial communities present in the rhizospheric soils of progenitors and closely related species of modern sugarcane hybrids as they play critical roles in plant nutrition, biotic and abiotic stress tolerance, and growth and development (Baldani et al., 2002; Li et al., 2017).

16S rRNA sequence data revealed 6,202 OTUs assigned to different bacterial species colonizing the rhizosphere of different sugarcane species studied. Analysis of these OTUs showed that *S. sinense* rhizosphere has the most significant number of rhizobacterial communities compared to other related species studied here. These results were also confirmed by qPCR analysis. qPCR analysis results demonstrated that 16S rRNA gene copy numbers were observed highest in *S. sinense* and lowest in *S. spontaneum*. The highest *nifH* gene numbers were observed in *S. robustum* and lowest in *S. spontaneum*. Besides this, alpha diversity analysis also predicted the highest bacterial diversity in *S. sinense* samples. However, other sugarcane species also showed considerable species diversity. Phylum level distribution studies identified the dominance of Proteobacteria, Firmicutes, Actinobacteria, Bacteroidetes, Chloroflexi, Gemmatimonadetes, Planctomycetes, and Nitrospirae. Many of them were reported to be present in the sugarcane rhizosphere previously (Gao et al., 2019). Out of the total OTUs identified, 1,099 were from diazotrophs based on *nifH* gene data. The nifH gene sequence analysis helped to identify the top twenty genera (*Bradyrhizobium, Dechloromonas, Desulfovibrio, Stenotrophomonas*, Xanthobacter *Anthobacter, Anaeromyxobacter, Azospirillum, Pseudoacidovorax, Methylobacterium, Azoarcus, Paenibacillus, Ideonella, Beijerinckia, Paraburkholderia, Burkholderia, Ruficoccus, Geobacter, Sinorhizobium, Kosakonia*, and *Azotobacter*). Out of these 20 genera, most of the genera were found to fix nitrogen in sugarcane and other plants. Genus *Bradyrhizobium* is known to fix nitrogen in sorghum and sugarcane (de Matos et al., 2019; Hara et al., 2019). *Azotobacter* genus consists of seven different species and they are involved in atmospheric nitrogen fixation in different crops (Jiménez et al., 2011). Genera *Azotobacter* and *Beijerinckia* were studied for diazotrophic attributes in the early 1960s in rice and other cereal crops (Dobereiner, 1961; Pankievicz et al., 2019). Contrary to *Azotobacter*, the *Beijerinckia* genus is restricted mainly to the tropics, and its nitrogen fixation ability has been reported in various plants (Nassar et al., 2020). *Kosakonia* spp. fixes nitrogen on cucumber roots (Sun et al., 2018). Roots of switchgrass are inhabited with nitrogen-fixing bacteria that belong to the genera *Dechloromonas, Desulfovibrio, Azoarcus, Ideonella, Paraburkholderia*, and *Burkholderia* (Bahulikar et al., 2019). It is hard to identify and classify most bacteria in culture because of their morphological similarities. But, culture-independent methods, such as 16S rRNA sequencing, are highly efficient, cost-effective and provide accurate identification and classification of rhizobacteria. Overall, the dominant genera identified in this study are known to fix atmospheric nitrogen, facilitating plant growth.

In the present study, we observed a few taxa, such as *Proteobacteria, Acidobacteria, Actinobacteria, Firmicute, Sphingomonas, Bradyrhizobium*, and *Gemmatimonas*, were dominant with *Bacillus, Pseudomonas, Bradyrhizobium, Burkholderia*, and *Rhizobium* as leading genera. In our previous studies, we observed the occurrence of *Proteobacteria, Acidobacteria, Actinobacteria, Firmicute, Bacteroidetes, Sphingomonas, Bradyrhizobium, Bryobacter*, and *Gemmatimonas* in the sugarcane rhizosphere (Solanki et al., 2018, 2020). Some genera, such as *Bacillus, Pseudomonas*, and *Burkholderia*, known for their plant-growth-promoting and nitrogen-fixing properties, were found to be enriched in the sugarcane rhizosphere (Li et al., 2017; Malviya et al., 2019; Singh et al., 2020). Community composition analysis of 16S rRNA sequence data helped to track phylum and genus level distribution of rhizobacteria among different sugarcane species studied. Interestingly, we observed the presence of a few genera, namely *Streptococcus, Rhodanobacter, Anaeromyxobacter*, and *Prevotella*, shared among all the species studied here. The members of these genera were found commonly in soil and can colonize crop plants. From the previous reports, it appears that *Rhodanobacter, Anaeromyxobacter*, and *Prevotella* species were isolated from different soil and plant sources, and they were found to have *nifH* gene and nitrogen fixation abilities (Igai et al., 2016; Espenberg et al., 2018). Thus, we believe that more characterization of these bacteria colonizing the sugarcane rhizosphere will be beneficial for developing bio-based crop products to improve sugarcane crop productivity. Diversity among these nitrogen-fixing bacteria was revealed by alpha and beta diversity analyses. The top genera with the highest abundance were found to be *Bacillus, Desulfovibrio, Xanthobacter, Anaeromyxobacter, Pseudoacidovorax, Azospirillum*, and *Methylobacterium*. Among these, *Bacillus* is a common bacterial diazotroph in sugarcane (Reis et al., 2007). *Desulfovibrio, Anaeromyxobacter, Azospirillum*, and *Xanthobacter* are nitrogen fixers in rice (Sessitsch et al., 2012; Bao et al., 2014; Yoneyam et al., 2017; Rosenblueth et al., 2018). Correlation results showed that among all bacteria, genera *Bacillus, Pseudomonas, Massilia, Pseudarthobacter*, and *Streptomyces* correlate positively with various soil parameters. Genera *Bacillus, Pseudomonas*, and *S*treptomyces are well-known for plant growth and plant disease suppression activities in sugarcane and other crops (Wang et al., 2019; Amna et al., 2020; Chandra et al., 2020; Jiao et al., 2021). Genus *Massilia* is a significant group of rhizosphere and root colonizing bacteria of many plant species (Ofek et al., 2012). Genus *Pseudarthobacter* plays an essential role in biodegradation (Gupta et al., 2020; Chen et al., 2021). Genera *Azotobacter and Ideonella* revealed a positive correlation with ${\text{NH}}_{4}^{+}$N, and several previous reports also demonstrated that these bacteria could fix atmospheric nitrogen efficiently and enhance the crop yield (Bhromsiri and Bhromsiri, 2010; Aasfar et al., 2021). Diazotroph *Anaeromyxobacter* correlated positively with ${\text{NH}}_{4}^{+}$N and recently reported that *Anaeromyxobacter* enhances the nitrogen-fixing activity of paddy soils (Masuda et al., 2020). *Desulfovibrio* had a positive correlation with available ${\text{NO}}_{3}^{-}$N. *Desulfovibrio* is known for its sulfur-reducing property and plays a particular and significant role in the fixation of biological nitrogen (Mistry et al., 2022). On the contrary, soil physiochemical, such as OM, showed a negative correlation with genera *Desulfarculus* and *Anabaena*, while ${\text{NH}}_{4}^{+}$N showed a negative correlation with genera *Methylobacter* and *Desulfarculus*. It may be due to a decrease of soil nutrients in the sugarcane rhizosphere that increases microbial completion (Jones et al., 2018; Solanki et al., 2020).

## Conclusions

The sugarcane species studied here showed a significant number of N-fixing rhizobacteria, which strengthens the contention that exploring rhizosphere bacteria may help to develop a sustainable low resource input sugarcane crop production system, particularly, for meeting its N requirement from atmospheric nitrogen fixation. Substantial genetic variation for rhizobacteria, such as diazotroph communities, exists among different progenitors and closely related species of modern cultivated sugarcane hybrids. However, considering the vast natural habitats of these wild species spanning tropics and subtropics, similar studies using accessions sourced from different locations and environmental conditions will significantly advance our understanding of sugarcane rhizobiome. Future research should also focus on *Saccharum* species introgression breeding and isolation and practical application of beneficial PGPRs, especially diazotrophs. Filling the significant knowledge gap on microbiota and sugarcane interactions is critical for exploiting these beneficial microbes for sustainable sugarcane agriculture.

## Data Availability Statement

The datasets presented in this study can be found in online repositories. The names of the repository/repositories and accession number(s) can be found in the article/Supplementary Material.

## Author Contributions

MM and C-NL conceived and designed the experiments and performed the experiments. MM, C-NL, and MS analyzed the data. ZW, ACS, KV, AS, RS, PS, and ED contributed to reagents/materials/analysis tools. Y-RL contributed to project administration. C-NL, QN, X-PS, and Y-RL contributed to funding acquisition. MM, MS, and PL contributed in writing-original draft and revision. All authors contributed to the article and approved the submitted version.

## Funding

This work was supported by grants from the Guangxi Innovation Teams of Modern Agriculture Technology (No. gjnytxgxcxtd-2021-03-01 to Y-RL), National Natural Science Foundation of China (Nos. 31701489 to QN, 31801288 to C-NL, and 31901594 to X-PS), the Natural Science Foundation of Guangxi Province (Nos. 2019GXNSFDA185004 and 2021GXNSFAA196041 to C-NL), and Guangxi Academy of Agricultural Sciences Fund (Nos. Guinongke 2021YT09 and Guinongke 2021JM01 to C-NL).

## Conflict of Interest

The authors declare that the research was conducted in the absence of any commercial or financial relationships that could be construed as a potential conflict of interest.

## Publisher's Note

All claims expressed in this article are solely those of the authors and do not necessarily represent those of their affiliated organizations, or those of the publisher, the editors and the reviewers. Any product that may be evaluated in this article, or claim that may be made by its manufacturer, is not guaranteed or endorsed by the publisher.
