# Supplementary material for: High-Throughput Sequencing-Based Analysis of Rhizosphere and Diazotrophic Bacterial Diversity Among Wild Progenitor and Closely Related Species of Sugarcane (Saccharum spp. Inter-Specific Hybrids)
Source: Front Plant Sci. 2022 Feb 24;13:829337. doi: 10.3389/fpls.2022.829337 (PMC8908384; doi:10.3389/fpls.2022.829337)
Supplement: Supplementary file 1 [file Table_1.DOCX]

**Supplementary materials**

**Table S1: The optimization sequence is the data used for subsequent cluster OTU and species information analysis of 16S rRNA**

| Sample name | Optimize the sequence | | | | | |
| --- | --- | --- | --- | --- | --- | --- |
|  | effective sequence number | optimization sequence number | base number | GC content (%) | average length | range |
| BRS | 46822 | 35489 | 14647201 | 55.756 | 413 | 350 –> 546 |
| PRS | 46829 | 38463 | 15875792 | 56.132 | 413 | 350 –> 444 |
| RRS | 47569 | 39589 | 16346082 | 56.119 | 413 | 406 –> 455 |
| SRS | 45328 | 35075 | 14457650 | 55.486 | 412 | 405 –> 573 |
| URS | 46907 | 37207 | 15346868 | 55.741 | 412 | 350 –> 444 |

**Table S2: The optimization sequence is the data used for subsequent cluster OTU and species information analysis of *nif*H**

| Sample name | Optimize the sequence | | | | | |
| --- | --- | --- | --- | --- | --- | --- |
|  | effective sequence number | optimization sequence number | base number | GC content (%) | average length | range |
| BRS | 36288 | 19751 | 7115998 | 63.246 | 360 | 208 –>413 |
| PRS | 36292 | 23809 | 8506893 | 62.513 | 357 | 211 –> 418 |
| RRS | 36434 | 22344 | 8029747 | 63.698 | 359 | 203 –> 413 |
| SRS | 36888 | 18350 | 6469064 | 61.150 | 353 | 200 –> 410 |
| URS | 36283 | 23393 | 8315569 | 62.663 | 355 | 202 –> 413 |

**Supplementary figures:**

**
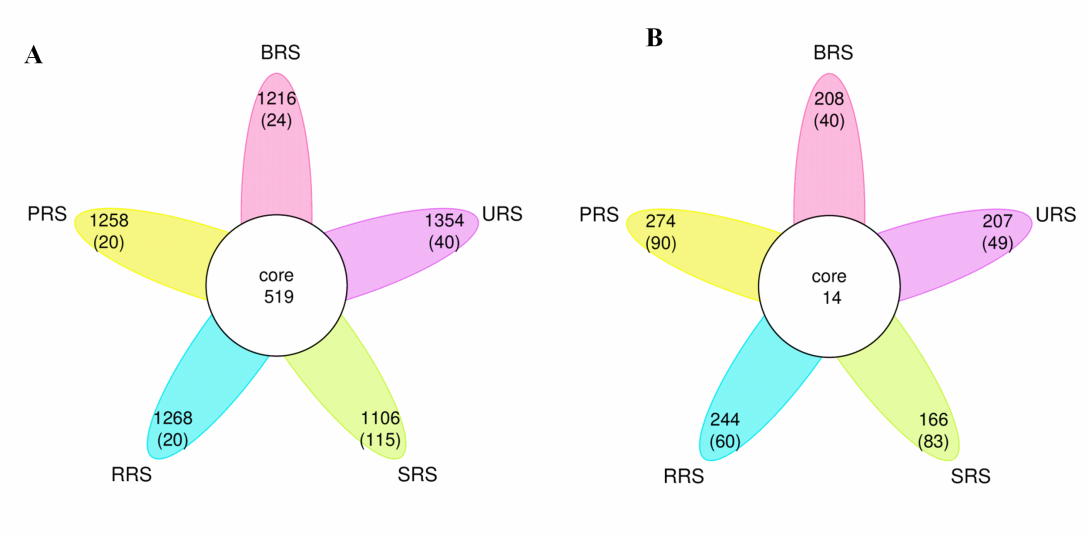
**

**Figure S1**. Venn diagram showing the OTUs of all sugarcane species (A) 16S rRNA and (B) *nifH* gene. *S*. *officinarum* L. cv Badila (BRS), *S*. *barberi* Jesw.cv pansahi (PRS), *S. robustum* (RRS), *S. spontaneum* (SRS), and *S. sinense* Roxb.cv. Uba (URS).

**Figure S2.** Principle component analysis **(A) 16S rRNA (B) *nifH* gene**

Different Points of color or shape represent groups of samples under different environments conditions, and the scale of horizontal and vertical axes is relative distance

**Figure S3.** Alpha diversity Shannon, Simpson and chao index of all sugarcane species (A) 16S rRNA and (B) nifH gene

**Figure S4. Star analysis showing the top 10 genus with the highest abundance (A) 16S rRNA** and **(B) *nifH* gene**

**Figure S5**. Beta diversity analysis to estimate the dissimilarity and similarity of 16S bacterial communities and composition among different samples. (a) Principal coordinated analysis (PCoA) derived from dissimilarity matrix of weighted UniFrac distance. (b) Multi-sample differential matrix heat map weighted UniFracbased cluster analysis of bacterial community composition among different samples. *S*. *officinarum* L. cv Badila (BRS), *S*. *barberi* Jesw. cv pansahi (PRS), *S.* *robustum* (RRS), *S. spontaneum* (SRS), and *S. sinense* Roxb. cv. Uba (URS).

**Figure S6**. Beta diversity analysis to estimate the dissimilarity and similarity of *nifH* bacterial communities and composition among different samples. (a) Principal coordinated analysis (PCoA) derived from dissimilarity matrix of weighted UniFrac distance. (b) Multi-sample differential matrix heat map weighted UniFracbased cluster analysis of bacterial community composition among different samples**.** *S*. *officinarum* L. cv Badila (BRS), *S*. *barberi* Jesw. cv pansahi (PRS), *S.* *robustum* (RRS), *S. spontaneum* (SRS), and *S. sinense* Roxb. cv. Uba (URS)
